# Aneurysmal subarachnoid haemorrhage: Effect of CRHR1 genotype on mental health-related quality of life

**DOI:** 10.1038/s41598-020-57527-4

**Published:** 2020-01-20

**Authors:** Artur Vetkas, Ele Prans, Sulev Kõks, Tõnu Rätsep, Toomas Asser

**Affiliations:** 1 Tartu University Hospital, Tartu, Estonia; 2 Tartu University, Tartu, Estonia; 3 Centre for Molecular Medicine and Innovative Therapeutics, Murdoch University, Perth, WA, Australia; 4 The Perron Institute for Neurological and Translational Science, Perth, WA Australia

**Keywords:** Predictive markers, Stroke

## Abstract

Quality of life (QoL) disturbances are common after aneurysmal subarachnoid hemorrhage (aSAH) both in physical and mental health domains and their causes are not clearly understood. Corticotropin-releasing hormone receptor 1 (CRHR1) is involved in stress reactivity and development of mental health disturbances after negative life-events. We performed a retrospective cohort study of long-term QoL outcomes among 125 surgically treated aSAH patients (2001–2013). QoL was assessed with Short Form Health Survey (SF-36) and compared to an age and gender matched general population. Genotyping of CRHR1 single nucleotide polymorphisms was performed (Rs7209436, Rs110402, Rs242924) and their effect on QoL scores was explored. aSAH patients experienced a reduced quality of life in all domains. CRHR1 minor genotype was associated with higher SF-36 mental health (OR = 1.31–1.6, p < 0.05), role-emotional (OR = 1.57, p = 0.04) and vitality scores (OR = 1.31–1.38, p < 0.05). Association of all studied SNP’s with vitality and Rs242924 with mental health scores remained statistically significant after Bonferroni correction. Mental quality of life scores were associated with physical state of patients, antidepressant history and CRHR1 genotype. Predisposition to mental health disturbances after stressful life-events might be associated with reduced mental QoL after aSAH and selected patients could be provided advanced counselling in the recovery phase.

## Introduction

Aneurysmal subarachnoid haemorrhage (aSAH) causes long-term morbidity and leads to reduced quality of life (QoL)^1^. Incidence of aSAH is around 7.9 per 100 000 patient years and is showing a trend of decrease^2,3^. More patients survive the subacute phase and in the long-term almost two-thirds of them are functionally independent^4^. Despite survival rates improving up to 65% and physical disability decreasing among survivors, psychosocial outcomes after aSAH remain to be notably poor since up to 55% of patients report reduced quality of life years after the haemorrhage^5^. Up to a half of aSAH patients have mental health complaints, including depression and anxiety^6,7^. Only one third of the patients resume the same work^8^.

The cause of these changes has not been explored, although genetic background may be involved in the development of psychosocial impairments. Genes that regulate the function of the stress response system are probable moderators of the effect that adverse life events have on development of long-term mental health disturbances^9,10^. Corticotropin-releasing hormone (CRH) is one of the main stress mediators in the central nervous system and plays a role in the etiology of emotional disorders^11,12^. Corticotropin-releasing hormone receptor 1 (CRHR1) genotype has been repeatedly associated with emotional disturbances and response to antidepressant treatment^13–15^. Effect of CRHR1 in major depression is moderated by a history of negative life events^16^ and CRHR1 genotype is associated with cortisol reactivity to stress^17–19^. The substantial reduction in mental health related QoL after aSAH associated with neuroendocrine dysfunction has been previously reported^20^. Emotional health disturbances are connected to quality of life disturbances and explain 23–47% of QOL score reductions in aSAH^21^.

Few articles have been published on the genetic background of QoL disturbances after aSAH and none have studied the effect of the hypothalamic–pituitary–adrenal (HPA)-system^22^. We hypothesize that CRHR1 genotype will influence the mental component of quality of life after aSAH.

## Methods

**Figure 1:**
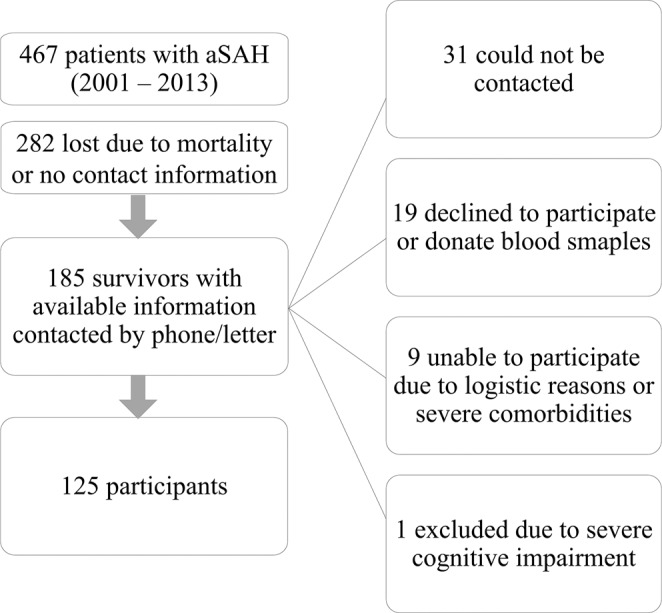
Study Flow Diagram.

We performed a retrospective cohort study of long-term outcome in a group of aSAH survivors (n = 125) surgically treated from January 2001 to November 2013 in a university clinic. All patients diagnosed with aSAH based on medical records during this period (n = 467) were included in the study (spontaneous ICH and other SAH causes were excluded). We identified 185 survivors with available contact information, who were proposed to participate in the study. Exclusion criteria and selection protocol are presented in Fig. 1. Eventually the studied group consisted of 125 patients. Blood samples were collected after the interview by the physician. Informed consent was obtained from all participating individuals and all procedures performed in this study involving human participants were in accordance with the ethical standards and the latest Helsinki declaration. Tartu University ethics committee approval 214/T-2/2012 was given for performing this study.

### General management

All patients were admitted during the acute phase of the disease. The diagnosis of aSAH was confirmed by computed tomography (CT) or lumbar puncture, and aneurysm location was assessed by CT-angiography or digital subtraction angiography. All patients were managed according to general guidelines and our protocol is previously published^21^. Patients were initially treated in a neurointensive care unit. Almost all patients were acutely operated upon, preferably via a pterional approach, and the aneurysms were clipped using standard microsurgical techniques. Endovascular procedures were preferentially performed in a separate institution during this time and due to this our study includes a series of clipped patients.

### Procedure

Clinical variables, including Hunt and Hess grade (HH)^23^, which is a grading system designed to predict prognosis and outcome in aSAH, were recorded in medical histories during admission. Remaining data was collected during the follow-up evaluation, when patients were interviewed in person with a structured questionnaire. Patient clinical recovery was evaluated according to the modified Rankin Score (mRS)^24^. Patients were also questioned about treatment for emotional disorders after aSAH, comorbidities, education and social living situation (living with family/someone else or alone). Short Form Health Survey (SF-36) was used to assess the health-related quality of life (HRQoL).

SF-36 is widely used in clinical outcome research and is a validated instrument to assess general QoL. SF-36 consists of 36 questions and measures eight scales: physical functioning, role-physical, bodily pain, general health, vitality, social functioning, role-emotional, and mental health^25,26^. SF-36 scores are transformed by assignment of predefined weights to the different items and calculated separately for each scale. Results can range from 0 (low QoL) to 100 (high QoL). Component scores are separately calculated for mental and physical health. SF-36 questionnaire has been added as Supplementary Material. Data of the study group was compared with corresponding values from an age-matched and gender-matched group of general population (996 subjects) obtained from respondents of a health survey of 1,989 individuals.

### Genotyping

The genomic DNA was extracted from venous blood samples in 4 ml EDTA containing vaccuettes by using the standard salting-out method. The EDTA tubes were stored at −20C until DNA extraction. Isolated DNA was dissolved in Tris-EDTA (TE) buffer. The purity and concentrations of the DNA were measured by a spectrophotometer (NanoDrop, ND-1000). The gDNA samples were aliquotted and stored at −80C until usage^27^. Genotyping of marker single nucleotide polymorphisms (SNP) rs7209436, rs110402, rs242924 and rs242939 was carried out by using TaqMan SNP Genotyping Assay (Applied Biosystems, Foster City, CA, USA), which is a multiplex endpoint assay that detects variants of a single nucleic acid sequence. PCR reactions were run on the ViiA7 instrument (Applied Biosystems, Foster City, CA, USA) by using the following cycling parameters: after the first step at 95C for 10 minutes, 40 cycles of denaturation at 92C for 15 seconds and extension at 60C for 1 minute. Genomic DNA (20 ng/ul) was amplified in a total volume of 10 ul containing 1× Amplification Master Mix (Applied Biosystems, Foster City, CA, USA) and 1× probe. Genotypes were analysed by using the allelic discrimination function of the system (Table 1).

**Table 1:** CRHR1 allele distribution (n = 125).

SNP	Genotype (n)			Minor allele (n)	Major allele (n)
Rs7209436	C/C (34)	C/T (69)	T/T (22)	T (91)	C (103)
Rs110402	G/G (29)	A/G (68)	A/A (28)	A (96)	G (97)
Rs242924	G/G (31)	G/T (67)	T/T (27)	T (94)	G (98)

Abbreviations: CRHR1 - corticotropin-releasing hormone receptor 1, SNP– single nucleotide polymorphism.

### Statistics

Student’s t-test was used to determine the associations between the subscale scores of the questionnaires and clinical/sociodemographic factors and compare the SF-36 mean scores of the patients with age and gender matched general population. All continuous variables were controlled for normality using Shapiro-Wilk’s W test. Beta-binomial regression analysis was performed to describe the association of CRHR1 genotype with SF-36 scale scores and calculate odds ratios. In the SNP analysis we chose between additive/dominant/recessive model based on the AIC (Akaike information criterion) of the unadjusted model. In the analysis of SF-36 results, an odds ratio (OR) higher than 1, indicates a better quality of life in the respective group (recessive model – minor allele homozygote; dominant model – major allele homozygote; additive model – OR for heterozygotes, which is multiplied in case of minor allele addition); odds ratio lower than 1 indicates a reduced outcome in SF-36 scales. More precisely, OR shows what is the probability of receiving a higher score in the selected scale by 1 point (1 point bring equal to 5 points in physical functioning, vitality, general health scales; 25 points in role-physical scale; about 11 points in pain scale; about 33 points in role-emotional scale, 12,5 points in social functioning scale and about 8 points in emotional wellbeing scale)^28,29^. Results were considered significant if p < 0.05. The p-values that survived the Bonferroni correction are marked in bold. Pearson’s correlation and multiple logistic regression models were used to study the impact of genotype (frequency of minor alleles), sociodemographic and clinical factors on SF-36 scores. Statistical analysis was performed with Stata 14.2 (StataCorp LLC) and SPSS 24 (IBM).

## Results

Patient and aSAH characteristics are presented in Table 2. Most were female (70%, n = 88) and the mean age at the time of the hemorrhage was 54 years (SD ± 13; range 24–82 years). The mean time between initial admission and the study was 4 years (SD ± 2.8; range 1–13 years). 51 patients (41%) were evaluated later than 3 years from ictus. Most of the patients (78%) had more than 10-years of education. Only 1 patient had a previous diagnosis of depression based on the medical histories available from 2009. The mean age of the patients at the time of follow-up was 58 years (SD ± 12, range 26–82). 78% (n = 97) of the patients were living with family or somebody else. 41% (n = 51) of the patients required daily help. The most common comorbidities were hypertension (67%), joint pain (14%) and diabetes (7%). 24% (n = 30) saw a psychologist or psychiatrist and 38% (n = 30) used antidepressants during recovery. There was no statistically significant difference between minor and major CRHR1 genotypes and sociodemographic characteristics.

**Table 2:** Patient (n = 125) and aSAH characteristics.

Characteristic		N	%
Male		37	30
Female		88	70
Hunt Hess score	1	17	14
	2	66	53
	3	23	18
	4	14	11
	5	5	4
Aneurysm location	ICA	40	32
	AcomA	44	35
	MCA	22	18
	ACA	8	6
	BA	9	7
	VA	2	2
Intracerebral haemorrhage		22	18
Symptomatic vasospasm		34	27
Hydrocephalus	acute	43	34
	chronic	14^*^	11
Modified Rankin Score	0	4	3
	1	7	6
	2	57	46
	3	49	39
	4	8	6

Abbreviations: ICA internal carotid artery, AcomA anterior communicating artery, MCA middle cerebral artery, ACA anterior cerebral artery, BA basilar artery, VA vertebral artery, aSAH aneurysmal subarachnoid haemorrhage. ^*^These 14 patients required ventriculoperitoneal shunting after aSAH.

Of the patients, 55% (n = 68) had a mRS score of 0–2 and 38% (n = 48) had a score of 3. mRS score was worse among women (2.5 (SD = 0.8) vs 2.1 (SD = 0.8), p = 0.019) and those requiring daily help (3.1 (SD = 0.5) vs 2.0 (SD = 0.7), p < 0.001).

### Quality of life scores

Quality of life scores of patients measured with SF-36 were significantly lower than the general population scores on all scales, except mental health (Table 3). Physical health scores and role limitation scores due to physical and emotional problems were affected the most. The mean SF-36 summary measures were: physical health component score (PCS-36) - 43 (SD ± 9.6) and mental health component score (MCS-36) - 48.6 (SD ± 9.4).

**Table 3:** Short Form Health Survey 36 results among patients and gender/age matched general population.

SF-36 scales	Mean aSAH (n = 125)	SD aSAH	Mean population (n = 996)	SD population	p
Physical Functioning	62.8	25.9	79	25.8	<0.001
Role-Physical	38	41.4	71.4	39.1	<0.001
Bodily Pain	66.2	27.7	72.6	26.4	0.008
General Health	48.6	21.4	56.3	19.2	<0.001
Vitality	51.3	20	55	18.9	0.03
Social Functioning	72.1	24.5	77.4	28.6	0.01
Mental Health	67.7	17.9	69.4	17.8	0.31
Role-Emotional	53.1	42	76.3	36.8	<0.001

Abbreviations: SF-36 - Short Form Health Survey 36, aSAH – aneurysmal subarachnoid haemorrhage, SD - standard deviation, p – P value.

Being older than 55 years old at ictus was associated with a worse physical functioning score (mean 54 (SD ± 25.4) vs 68 (SD ± 25), p = 0.003) and a worse PCS-36 score (mean 40.6 (SD ± 9.4) vs 44.6 (SD ± 9.5), p = 0.024).

Being female was associated with a worse physical functioning score (mean 56.9 (SD ± 26.4) vs 76.6 (SD ± 19.1), p < 0.001); role-physical score (mean 32.4 (SD ± 38.6) vs 51.4 (SD ± 45.6), p = 0.019); mental health score (mean 64.9 (SD ± 18.9) vs 74.1 (SD ± 13.7), p = 0.08) and a worse PCS-36 score (mean 41.6 (SD ± 9.2) vs 46.8 (SD ± 9.5), p = 0.005).

Having more than 3 years from aSAH to evaluation was associated with a worse physical functioning score (mean 58.3 (SD ± 26.7) vs 67.4 (SD ± 24.5), p = 0.048); a worse general health score (mean 43 (SD ± 20.5) vs 54.2 (SD ± 21.1), p = 0.003); and a worse PCS-36 score (mean 40.8 (SD ± 8.8) vs 45.4 (SD ± 9.9), p = 0.008). The difference in physical functioning score became statistically insignificant after adjustment for age.

Hypertension was associated with a worse physical functioning score (mean 58.9 (SD ± 25) vs 70.9 (SD ± 26.3), p = 0.018). Diabetes was also associated with a worse physical functioning score (mean 40 (SD ± 24.1) vs 64.6 (SD ± 25.4), p = 0.016). Having joint pains or rheumatoid arthritis was associated with a worse role-physical score (mean 18.1 (SD ± 26.9) vs 41.5 (SD ± 42.8), p = 0.004).

mRS score was negatively correlated to all SF-36 scales with −0.62 for physical functioning, −0.47 for role-physical, −0.45 for general health and −0.45 for role-emotional (p < 0.001). Correlations with mental health, vitality, social functioning scales and pain were below −0.4 (p < 0.001).

### Association of CRHR1 genotype with SF-36 outcomes

In beta-binomial regression analysis we explored the association of CRHR1 genotype with SF-36 quality of life scores (Table 4). CRHR1 minor alleles (rs7209436, rs110402, and rs242924) were associated with higher mental health scores (OR = 1.31 − 1.6, p < 0.05) in additive and recessive models; and higher vitality scores (OR = 1.31 − 1.38, p < 0.05) in the additive model. Rs7209436 minor alleles were associated with a higher role-emotional score (OR = 1.57, 95% CI, 1.01–2.44, p = 0.044) in the additive model and rs110402 major alleles with a lower role-emotional score (OR = 0.43, 95% CI, 0.21–0.87, p = 0.019) in the dominant model. The results remained statistically significant after adjustment for gender, neurological state at admission (HH), patient age and time from aSAH to evaluation.

**Table 4:** Association of genotype with Short Form Health Survey 36 scales (only statistically significant results are reported).

SNP	Allele	Model	OR	95% CI	p	OR*	95% CI*	p*
**Mental health**								
Rs7209436	Minor	Additive	1.31	1.07–1.6	0.009	1.31	1.07–1.6	0.009
Rs110402	Minor	Additive	1.29	1.06–1.57	0.011	1.26	1.04–1.54	0.019
Rs242924	Minor	Recessive	1.6	1.14–2.24	**0.006**	1.59	1.14–2.22	**0.007**
**Vitality**								
Rs7209436	Minor	Additive	1.38	1.13–1.7	**0.002**	1.38	1.13–1.69	**0.002**
Rs110402	Minor	Additive	1.31	1.07–1.6	**0.008**	1.31	1.07–1.6	0.009
Rs242924	Minor	Additive	1.33	1.09–1.62	**0.005**	1.32	1.08–1.62	**0.006**
**Role-emotional**								
Rs7209436	Minor	Additive	1.57	1.01–2.44	0.044	1.53	0.98–2.4	0.063
Rs110402	Major	Dominant	0.43	0.21–0.87	0.019	0.44	0.22–0.91	0.026

Abbreviations: SNP – single nucleotide polymorphism, OR-odds ratio, CI-confidence interval, p –P-value. *-values adjusted for sex, Hunt Hess score, patient age and time of evaluation from aSAH. P-values that survived the Bonferroni correction are marked with bold.

Effect of CRHR1 genotype on mental health (Rs242924) and vitality scales (all SNP’s) remained statistically significant after Bonferroni correction for multiple comparisons. Homozygotes for Rs242924 minor allele had higher mental health scores (OR = 1.6, 95% CI, 1.14–2.24, p = 0.007). Homozygotes for minor alleles of Rs7209436 (OR = 1.9, p = 0.002), Rs110402 (OR = 1.69, p = 0.008) and Rs242924 (OR = 1.77, p = 0.005) had higher vitality scores.

Patients with more minor alleles of rs7209436, rs110402, and rs242924 had higher mental QoL scores. Homozygotes for minor allele of Rs242924 had a higher mental health score compared to major allele carriers - 76 vs 66. Homozygotes for minor alleles of Rs7209436, Rs110402 and Rs242924 had higher vitality scores (60 vs 43; 58 vs 44; and 58 vs 44, respectively). Same trend is observable for other alleles in Table 5. rs242939 did not show a statistically significant association with SF-36 scores. TAT-haplotype, formed by the three minor alleles, did not show any statistically significant effect on the quality of life scores.

**Table 5:** Average scores of SF-36 QoL scales associated with CRHR1 genotype according to major and minor alleles.

Genotype	Mental health	Vitality	Role-emotional
**Rs7209436**			
MM	63.5 ± 24.0	43.4 ± 15.3	41.2 ± 40.5
mM	67.2 ± 18.5	52.5 ± 21.4	55.6 ± 43.1
mm	75.5 ± 15.4	59.5 ± 17.6	63.6 ± 36.1
**Rs110402**			
MM	63.3 ± 17.4	44.0 ± 15.1	36.8 ± 39.5
mM	66.6 ± 18.5	51.7 ± 22.1	55.9 ± 42.6
mm	74.7 ± 14.9	57.9 ± 16.3	63.1 ± 38.2
**Rs242924**			
MM	65.9 ± 17.9	43.7 ± 15.3	n/a
mM	65.3 ± 18.3	52.0 ± 21.9	n/a
mm	75.7 ± 14.2	58.1 ± 16.6	n/a

Abbreviations: n/a – not associated. MM – homozygote for major allele, mM – heterozygote, mm – homozygote for minor allele.

### Factors influencing mental QoL

The best multiple regression analysis models for SF-36 scales associated with CRHR1 genotype (Table 6) included mRS score, physical health component score, antidepressant usage history and CRHR1 genotype (number of minor alleles), with R^2^ = 0.36 for role-emotional, R^2^ = 0.32 for vitality and R^2^ = 0.3 for mental health score, all p < 0.001 (Table 6). Minor allele count of Rs110402 was associated with role-emotional score (β = 0.15, p = 0.044) and of Rs7209436 with vitality score (β = 0.23, p = 0.003). PCS-36 score was associated with role-emotional (β = 0.45, p < 0.001), vitality (β = 0.5, p < 0.001) and mental health scores (β = 0.26, p = 0.005). mRS score was associated with role-emotional (β = −0.19, p = 0.032) and mental health scores (β = −0.23, p = 0.013). Antidepressant usage history was associated with mental health score (β = −0.23, p = 0.007). Sociodemographic factors, aneurysm location and comorbidities were not associated with SF-36 mental scale outcomes in multiple regression analysis.

**Table 6:** Multiple regression for SF-36 scales associated with CRHR1 genotype.

Variables	B	SE	β	p-value	R^2^
**Role-Emotional**					
PCS-36	1.96	0.37	0.45	<0.001	0.36
mRS	−15.56	7.17	−0.19	0.032	
Rs110402	9.43	4.64	0.15	0.044	
**Vitality**					
PCS-36	1.04	0.16	0.5	<0.001	0.32
Rs7209436	6.85	2.3	0.23	0.003	
**Mental Health**					
PCS-36	0.48	0.17	0.26	0.005	0.3
Antidepressants	−8.41	3.06	−0.23	0.007	
mRS	−8.13	3.23	−0.23	0.013	

## Discussion

In this retrospective cohort study, we describe the effect of CRHR1 gene polymorphisms on long-term quality of life outcomes after aSAH measured with SF-36 questionnaire. Beta-binomial regression analysis showed that CRHR1 genotype (rs7209436, rs110402, and rs242924) significantly affected mental quality of life outcomes after aSAH in studied patients. CRHR1 minor genotype carriers had higher quality of life scores in mental health, role-emotional and vitality scales, compared to the more common alleles. Rs110402 major genotype increased the risk of a worse score in role-emotional scale. Results remained statistically significant after adjustment for gender, neurological state at admission (HH), patient age and time from aSAH to evaluation. Effect of CRHR1 genotype on mental health (Rs242924) and vitality scales (all SNP’s) remained statistically significant after Bonferroni correction.

In our study, aSAH patients scored significantly lower on all SF-36 scales when compared to age and gender matched general population, except mental health where the difference did not reach statistical significance. We describe a more pronounced effect of aSAH on the physical component of QoL. Age, female gender, time from aSAH and comorbidities were associated with worse physical scale and general health scores. Female gender was associated with worse mental health. All SF-36 scales were negatively correlated to mRS score. Compared to a Swedish population studied with SF-36 after ischemic stroke, aSAH patients scored higher on physical function, but lower in mental health scales^30^. When compared to a group of 4-year myocardial infarction survivors aged under 65 years the biggest difference presented in role-limitation scores, with aSAH patients scoring lower^31^. Our patient group scored somewhat lower in the mental health scale of SF-36 than the age and gender matched general population, but the difference did not reach statistical significance. Elsewhere we have shown that when studied with a validated emotional health survey (EST-Q), which is based on DSMIV and ICD-10 diagnostic criteria, the same patient group exhibited an almost 3 times higher prevalence of depression and anxiety symptoms as an age and gender matched population. SF-36 mental health scale score consists of five questions (24–26, 28, 30). The shortage of statistical difference in detailed analysis of mental health scale depended on a better result of the study population in question 30 (amount of time spent being happy in the last 4 weeks). Patients scored higher than the general population on this sole question – 51 (SD ± 25.3) vs 46.4 (SD ± 27.8), p = 0.03. When question 30 was excluded and the remaining four questions averaged, the patient group scored significantly lower than the general population – 71.8 (SD ± 19.5) vs 75.2 (SD ± 18.4), p = 0.03.

Role-emotional and vitality scores were substantially lower among aSAH patients and were significantly affected by CRHR1 genotype. Role-emotional scale shows the subjective limitations people add to daily activities due to perceived mental problems. Vitality scale is a measure of energy/fatigue and it is often reduced after severe illness^32^. Fatigue could present in patients due to a state of mental exhaustion from having to deal with the processes of rehabilitation and adaptation to a new situation.

The three scales of SF-36, that reflect on the mental component of quality of life, were affected by CRHR1 genotype. Other factors that influenced the results, based on the multiple regression analysis, were modified Rankin scale, PCS-36 score and history of antidepressant usage. Two thirds of the variables affecting mental QoL scores remained unknown. It was previously reported that depression and fatigue have a significant role in mental QoL score reductions measured with SF-36, explaining 42% to 47% of the variance^21^. Involvement of CRHR1 genotype in the present study might be explained by its association with mental health disease. CRHR1 receptor is important in regulation of HPA-axis reactivity to stress and its genotype moderates the risk of mental health disorders after stressful life events^16,33^, including severe illness and intensive care treatment. CRHR1 (rs1876831) genotype has been associated with post-traumatic stress disorder and depression symptoms in critical illness survivors^34^. This warrants for further research and separate evaluation of specific QoL modalities in patients after aSAH with focus on mental health.

Despite advances in diagnosis and early management of aSAH, outcomes for most patients remain suboptimal and long-term sequelae of the disease may be unrecognized. Quality of life in aSAH patients has been linked to both physical and mental health disturbances in the long-term^5,35–39^. Variations exist in the capacity and timing of recovery in different components of quality of life after aSAH, with emotional health requiring more time to improve^40^. It was reported that productivity losses related to aSAH reached £278.9 million in the UK in 2005^41^. Neuropsychological disturbances can affect the ability of patients to return to work^42,43^, but proper rehabilitation can lead to reintegration in the long term^44^. We suggest that role of CRHR1 genotype warrants more research as it could help provide optimal rehabilitation and neuro-psychological support for aSAH patients.

### Study limitations

Our study consisted of 125 subjects and was a retrospective design. A selection bias due to loss of follow up exists and is related to the longevity of the study, social factors and patient participation. Although, we describe an association of CRHR1 genotype with mental QoL, our study might be underpowered to draw certain conclusions. Our findings require replication in a larger cohort. It is unknown whether the studied SNP-s are functional, or they are in linkage disequilibrium with other regions. Despite mental health diagnosis being extractable from the national database, we do not know the full extent of previous emotional problems in our patient group. We lack information regarding the cognitive profile of the patients, but none of them had severe disabilities when interviewed. SF-36 is a generic questionnaire that measures multiple modalities of quality of life. It has a correlation around 0.7 with other QoL assessment tools^45–47^. Differences exist in results among QoL questionnaires and subscales exhibit ceiling and floor effects^30,48^. Cultural factors can also influence outcomes.

## Conclusion

Results of our study suggest that CRHR1 genotype could influence mental quality of life outcomes after aSAH. rs7209436, rs110402, and rs242924 minor genotype significantly increased SF-36 mental health, role emotional and vitality scale scores This effect might be explained by CRHR1 role in the pathogenesis of emotional disorders and stress reactivity. New and improved biomarkers are needed to predict, diagnose and treat the long-term consequences of aSAH. Such biomarkers could help identify patients who would benefit from early neuropsychological rehabilitation.

## Supplementary information

SF-36.

Tables.
