# Supplementary material for: Aneurysmal subarachnoid haemorrhage: Effect of CRHR1 genotype on mental health-related quality of life
Source: Sci Rep. 2020 Jan 20;10:724. doi: 10.1038/s41598-020-57527-4 (PMC6971041; doi:10.1038/s41598-020-57527-4)
Supplement: Supplementary file 2 — Tables. [file 41598_2020_57527_MOESM2_ESM.docx]

Table 1. CRHR1 allele distribution (n=125).

| SNP | Genotype (n) | | | | Minor allele (n) | | Major allele (n) |  |
| --- | --- | --- | --- | --- | --- | --- | --- | --- |
| Rs7209436 | C/C (34) | C/T (69) | T/T (22) | T (91) | | C (103) | | |
| Rs110402 | G/G (29) | A/G (68) | A/A (28) | A (96) | | G (97) | | |
| Rs242924 | G/G (31) | G/T (67) | T/T (27) | T (94) | | G (98) | | |

Abbreviations: CRHR1 - corticotropin-releasing hormone receptor 1, SNP– single nucleotide polymorphism.

Table 2. Patient and aSAH characteristics.

| Characteristic |  | N | % |
| --- | --- | --- | --- |
| Male |  | 37 | 30 |
| Female |  | 88 | 70 |
| Hunt Hess score | 1 | 17 | 14 |
|  | 2 | 66 | 53 |
|  | 3 | 23 | 18 |
|  | 4 | 14 | 11 |
|  | 5 | 5 | 4 |
| Aneurysm location | ICA | 40 | 32 |
|  | AcomA | 44 | 35 |
|  | MCA | 22 | 18 |
|  | ACA | 8 | 6 |
|  | BA | 9 | 7 |
|  | VA | 2 | 2 |
| Intracerebral haemorrhage |  | 22 | 18 |
| Symptomatic vasospasm |  | 34 | 27 |
| Hydrocephalus | acute | 43 | 34 |
|  | chronic | 14^*^ | 11 |
| Modified Rankin Score | 0 | 4 | 3 |
|  | 1 | 7 | 6 |
|  | 2 | 57 | 46 |
|  | 3 | 49 | 39 |
|  | 4 | 8 | 6 |

Table 3. Short Form Health Survey 36 results among patients and gender/age matched general population.

| SF-36 scales | Mean aSAH (n=125) | SD aSAH | Mean population (n=996) | SD population | p |
| --- | --- | --- | --- | --- | --- |
| Physical Functioning | 62.8 | 25.9 | 79 | 25.8 | < 0.001 |
| Role-Physical | 38 | 41.4 | 71.4 | 39.1 | < 0.001 |
| Bodily Pain | 66.2 | 27.7 | 72.6 | 26.4 | 0.008 |
| General Health | 48.6 | 21.4 | 56.3 | 19.2 | < 0.001 |
| Vitality | 51.3 | 20 | 55 | 18.9 | 0.03 |
| Social Functioning | 72.1 | 24.5 | 77.4 | 28.6 | 0.01 |
| Mental Health | 67.7 | 17.9 | 69.4 | 17.8 | 0.31 |
| Role-Emotional | 53.1 | 42 | 76.3 | 36.8 | < 0.001 |

Abbreviations: SF-36 - Short Form Health Survey 36, aSAH – aneurysmal subarachnoid haemorrhage, SD - standard deviation, p – P value.

Table 4. Association of genotype with Short Form Health Survey 36 scales (only statistically significant results are reported).

| **SNP** | **Allele** | **Model** | **OR** | **95% CI** | **p** | **OR*** | **95% CI*** | **p*** |  |
| --- | --- | --- | --- | --- | --- | --- | --- | --- | --- |
| **Mental health** | | | | | | | | | |
| Rs7209436 | Minor | Additive | 1.31 | 1.07-1.6 | 0.009 | 1.31 | 1.07-1.6 | 0.009 |  |
| Rs110402 | Minor | Additive | 1.29 | 1.06-1.57 | 0.011 | 1.26 | 1.04-1.54 | 0.019 |  |
| Rs242924 | Minor | Recessive | 1.60 | 1.14-2.24 | **0.006** | 1.59 | 1.14-2.22 | **0.007** |  |
| **Vitality** | | | | | | | | | |
| Rs7209436 | Minor | Additive | 1.38 | 1.13-1.7 | **0.002** | 1.38 | 1.13-1.69 | **0.002** |  |
| Rs110402 | Minor | Additive | 1.31 | 1.07-1.6 | **0.008** | 1.31 | 1.07-1.6 | 0.009 |  |
| Rs242924 | Minor | Additive | 1.33 | 1.09-1.62 | **0.005** | 1.32 | 1.08-1.62 | **0.006** |  |
| **Role-emotional** | | | | | | | | | |
| Rs7209436 | Minor | Additive | 1.57 | 1.01-2.44 | 0.044 | 1.53 | 0.98-2.4 | 0.063 |  |
| Rs110402 | Major | Dominant | 0.43 | 0.21-0.87 | 0.019 | 0.44 | 0.22-0.91 | 0.026 |  |

Abbreviations: SNP – single nucleotide polymorphism, OR-odds ratio, CI-confidence interval, p –P-value. *-values adjusted for sex, Hunt Hess score, patient age and time of evaluation from aSAH. P-values that survived the Bonferroni correction are marked with bold.

Table 5. Average scores of SF-36 QoL scales associated with CRHR1 genotype according to major and minor alleles.

| Genotype | Mental health | Vitality | Role-emotional |
| --- | --- | --- | --- |
| Rs7209436 | | | |
| MM | 63.5±24.0 | 43.4±15.3 | 41.2±40.5 |
| mM | 67.2±18.5 | 52.5±21.4 | 55.6±43.1 |
| mm | 75.5±15.4 | 59.5±17.6 | 63.6±36.1 |
| Rs110402 | | | |
| MM | 63.3±17.4 | 44.0±15.1 | 36.8±39.5 |
| mM | 66.6±18.5 | 51.7±22.1 | 55.9±42.6 |
| mm | 74.7±14.9 | 57.9±16.3 | 63.1±38.2 |
| Rs242924 | | | |
| MM | 65.9±17.9 | 43.7±15.3 | n/a |
| mM | 65.3±18.3 | 52.0±21.9 | n/a |
| mm | 75.7±14.2 | 58.1±16.6 | n/a |

Abbreviations: n/a – not associated. MM – homozygote for major allele, mM – heterozygote, mm – homozygote for minor allele.

Table 6. Multiple regression for SF-36 scales associated with CRHR1 genotype.

| **Variables** | **B** | **SE** | **β** | **p-value** | **R^2^** |  |
| --- | --- | --- | --- | --- | --- | --- |
| **Role-Emotional** | | | | | | |
| PCS-36 | 1.96 | .37 | 0.45 | <0.001 | 0.36 |  |
| mRS | -15.56 | 7.17 | -0.19 | 0.032 |  |  |
| Rs110402 | 9.43 | 4.64 | 0.15 | 0.044 |  |  |
| **Vitality** | | | | | | |
| PCS-36 | 1.04 | 0.16 | 0.5 | <0.001 | 0.32 |  |
| Rs7209436 | 6.85 | 2.3 | 0.23 | 0.003 |  |  |
| **Mental Health** | | | | | | |
| PCS-36 | 0.48 | 0.17 | 0.26 | 0.005 | 0.3 |  |
| Antidepressants | -8.41 | 3.06 | -0.23 | 0.007 |  |  |
| mRS | -8.13 | 3.23 | -0.23 | 0.013 |  |  |
